# Meta-analysis of diffusion tensor imaging studies shows altered fractional anisotropy occurring in distinct brain areas in association with depression

**DOI:** 10.1186/2045-5380-1-3

**Published:** 2011-09-27

**Authors:** Melissa L Murphy, Thomas Frodl

**Affiliations:** 1 Department of Psychiatry and Institute of Neuroscience, School of Medicine, Trinity College Dublin, University of Dublin, College Green, Dublin 2, Ireland

## Abstract

Fractional anisotropy anomalies occurring in the white matter tracts in the brains of depressed patients may reflect microstructural changes underlying the pathophysiology of this disorder. We conducted a meta-analysis of fractional anisotropy abnormalities occurring in major depressive disorder using voxel-based diffusion tensor imaging studies. Using the Embase, PubMed and Google Scholar databases, 89 relevant data sets were identified, of which 7 (including 188 patients with major depressive disorder and 221 healthy controls) met our inclusion criteria. Authors were contacted to retrieve any additional data required. Coordinates were extracted from clusters of significant white matter fractional anisotropy differences between patients and controls. Relevant demographic, clinical and methodological variables were extracted from each study or obtained directly from authors. The meta-analysis was carried out using Signed Differential Mapping. Patients with depression showed decreased white matter fractional anisotropy values in the superior longitudinal fasciculus and increased fractional anisotropy values in the fronto-occipital fasciculus compared to controls. Using quartile and jackknife sensitivity analysis, we found that reduced fractional anisotropy in the left superior longitudinal fasciculus was very stable, with increases in the right fronto-occipital fasciculus driven by just one study. In conclusion, our meta-analysis revealed a significant reduction in fractional anisotropy values in the left superior longitudinal fasciculus, which may ultimately play an important role in the pathology of depression.

## Introduction

Major depressive disorder (MDD) is one of the most common human diseases, with a lifetime prevalence of 16% and an annual incidence of 6.6% [1]. It is a major cause of long-term disability, with approximately 800,000 individuals worldwide dying each year as a result of suicide, a high proportion of whom have or had MDD [2]. The World Health Organization (WHO) estimates that more people die each year as a result of suicide than in all the armed conflicts worldwide [2]. Even with treatment, approximately 40% of patients do not respond to the first antidepressant prescribed, and 20% experience chronic depression [3]. Many theories exist regarding the pathophysiological basis of MDD, though it remains unresolved. However, recent studies have highlighted many interesting neuronal processes occurring concurrently in the brains of patients with MDD, and these processes may interact with one another to increase or decrease an individual's susceptibility to depression. MDD is believed to originate from a combination of a susceptible genotype, chronic stress and an adverse developmental environment, leading to alterations in the biochemistry, neuroplasticity and structure in the brain [3-7]. Recent advances in neuroimaging techniques have allowed us to study the microstructural changes occurring both prior to and as a result of this disorder.

Magnetic resonance diffusion tensor imaging (DTI) is a novel neuroimaging technique that can evaluate both the orientation and the diffusion characteristics of white matter (WM) tracts *in vivo *[8]. DTI is sensitive to the diffusion patterns of water molecules and produces a three-dimensional image of the brain as a function of this water diffusion [9]. By measuring the direction and magnitude of restricted tissue water motility (diffusion anisotropy), the orientation of WM tracts in the brain can be determined [10], allowing the investigator to assess microstructural changes occurring in response to individual genotypes and environmental factors. This water diffusion occurs in three dimensions and is represented by the three eigenvectors (λ1, λ2 and λ3), with the major eigenvector reflecting the direction of maximum diffusivity, thus revealing the orientation of that fibre tract [8]. Factors that cause reduced water motility include the parallel arrangement of adjacent WM fibres within bundles, myelination, axonal filaments and neurofibrils [8]. Fractional anisotropy (FA) is a scalar value between 0 and 1 that measures the directionality of this water diffusion and serves as an important index of structural connectivity [9,10]. Finally, using tractography or region of interest (ROI) analysis, the structural characteristics of WM bundles in an area of interest can be determined [8]. Reduced FA in the absence of gross pathological findings may represent microstructural abnormalities diminishing the integrity of the WM tracts [11].

Numerous studies conducted using DTI in psychiatric patients have found FA abnormalities in certain brain regions, suggesting that WM structural anomalies exist in diseases such as bipolar disorder [12,13], schizophrenia [14,15] and depression [16,17]. Recent DTI studies have suggested that there is a strong correlation between depression and reduced FA, with the nature of this relationship being a topic of great interest. A study comparing 13 patients with late-life depression to age-matched healthy controls found a reduction in FA in both the frontal and temporal lobes of depressed patients [10]. In addition, an inverse relationship was established between FA values and symptom severity [10]. Another recent study conducted in MDD patients using whole-brain DTI analysis found reduced FA in the left sagittal stratum, the right cingulate cortex and the posterior body of the corpus callosum, areas of the brain believed to play an important role in emotional regulation [18]. Importantly, reductions in FA have also been associated with early-life stress (ELS) in the form of disrupted mother-infant attachment and correlate with an increased risk of both anxiety and depression [11]. A study comparing 12 maternally deprived adult male macaques to 9 normally reared controls found significant reductions in FA in the anterior limb of the internal capsule in the maternally deprived macaques [11]. This is another brain region important in emotional regulation and is involved in the medial and basolateral limbic circuits [11]. Thus, disruption of this region may alter functional connectivity between the frontal and temporal lobes, conferring an increased risk of MDD [11]. Another study demonstrating the microstructural implications of ELS found significantly reduced FA within the genu of the corpus callosum among those exposed to high levels of ELS [19]. DTI represents the forefront of neuroimaging techniques in the characterisation of microstructural alterations occurring in the brain, both as an antecedent to and as a consequence of depression [9].

The aims of this meta-analysis were to identify whether and where FA is altered in patients with MDD compared to healthy controls and to examine the impact that disease variables such as severity, treatment and duration have on these parameters.

## Methods

### Inclusion studies

An extensive search of databases, including Google Scholar, Embase and PubMed was carried out using keywords including 'DTI + depression' and 'Diffusion tensor imaging + major depressive disorder/MDD'. Inclusion criteria were studies that (1) conducted voxel-based analysis of magnetic resonance imaging scans, (2) compared FA value differences between depressed patients and healthy controls, (3) reported whole-brain analysis in stereotactic coordinates and (4) produced results corrected for multiple comparisons. Exclusion criteria for the final meta-analysis were (1) studies of depression in the context of other diseases, such as Huntington's disease, Parkinson's disease, mania, bipolar disorder, HIV and traumatic brain injury (TBI); (2) studies that measured antidepressant efficacy in depression; (3) studies that used ROI or tractography analysis; (4) studies that used uncorrected methods of analysis; and (5) literature reviews. We also contacted authors for additional information on their studies. This resulted in seven studies' being included in the meta-analysis, and these are summarised in Table 1. However, 21 studies using DTI that investigated patients with MDD or ELS compared to controls were taken into account in our discussion. With respect to depression severity, different studies used different psychopathological measurements. These were translated into a standardised depression severity score whereby 0 indicates healthy, 1 indicates mild depression, 2 indicates medium depression, 3 indicates severe depression and 4 indicates very severe depression http://www.ids-qids.org/index2.html#table2. None of the studies of late-life depression met our inclusion criteria, and these studies are discussed separately.

**Table 1:** Demographic and clinical characteristics of the seven voxel-based diffusion tensor imaging studies included in our meta-analysis^a^

Characteristics	Kieseppä ***et al. ***[18]	Blood ***et al. ***[24]	Zhu ***et al. ***[58]	Wu ***et al. ***[25]	Korgaonkar ***et al. ***[60]	Jia ***et al. ***[26]	Abe ***et al. ***[59]
Number of patients	16	22	25	23	29	52	21
Number of controls	20	22	25	21	39	52	42
Mean age of patients, years	48.4	36.3	20.6	31.4	40.5	34.5	48.1
Mean age of controls, years	42	35.3	20.3	30.4	29.6	37.1	48
Patient sex, % female	87.5	54.5	60	65.5	58.7	51.9	47.6
Control sex, % female	50	54.5	60	56.5	53.8	54	47.6
Late-life depression	0	0	0	0	0	0	0
Major depressive disorder	1	1	1	1	1	1	1
Anxiety in patients (0 = no, 1 = yes)	NA	0	0	0	NA	0	0
Treatment, % patients receiving medication at time of study	81	31.8	0	0	0	0	90.5
Mean illness duration, years	14.1	NA	13.6	2.2	NA	3.3	6
Mean BDI score	26.3	NA	NA	NA	NA	NA	NA
Hamilton score	NA	NA	NA	21.8	19.1	23	9.2
IDS-SR score	NA	36	NA	NA	NA	NA	NA
Mean CES-D score	NA	NA	35.48	NA	NA	NA	NA
Illness severity	2	2	3	3	3	3	1

^a^BDI, Beck Depression Inventory; IDS-SR, Inventory of Depressive Symptoms-Self-Rated; CES-D, Center for Epidemiologic Studies Depression Scale. NA, not applicable.

### Meta-analysis of studies

Meta-analytical regional differences in FA values of WM tracts were calculated using mean and threshold probability procedures with Signed Differential Mapping (SDM) software http://www.sdmproject.com/. This software uses restricted maximum likelihood estimation of the variance with the reported peak coordinates to recreate maps of the positive and negative FA differences between patients and controls rather than just assessing the probability or likelihood of a peak [20]. This allowed us to accurately compare patients and controls without biasing the results towards brain regions that showed high variability between studies [21].

SDM converts DTI coordinates to Talairach space with cluster peaks from DTI studies represented on an SDM or MRIcron brain map http://www.cabiatl.com/mricro/mricron/main.html, highlighting areas of the brain where FA alterations reach significant values. Peak coordinates of FA differences between patients and controls are extracted from each data set. Peaks that are not statistically significant at the whole-brain level are excluded from these maps. This is done to ensure that the same statistical threshold throughout the brain is used within each study. Therefore, bias towards liberally thresholded brain regions is avoided, as it is not uncommon in neuroimaging studies for the statistical threshold of some ROIs to be more liberal than those in the rest of the brain. Next, a standard Talairach map of the differences in WM is recreated separately for each study by means of a Gaussian kernel that assigns higher values to the voxels closer to peaks. This includes (1) limiting voxel values to a maximum to avoid bias towards studies reporting various coordinates in close proximity and (2) reconstructing both increases and decreases in WM volume in the same map. Mean analysis, which calculates the mean value of each voxel, was carried out in our meta-analysis, with studies containing a larger sample size having more weight. Jackknife analysis was also carried out on the included studies to ensure that one study did not significantly affect our results and that the FA values obtained were highly replicable throughout all of the studies. Moreover, descriptive analyses of quartiles were used to find the actual proportion of studies reporting results in a particular brain region. Statistical significance was determined by using standard randomization tests, thus creating null distributions from which *P *values could be obtained directly [22]. We focus on results with *P *< 0.001 for significance in the group differences and *P *< 0.0002 for the meta-regression analysis. For meta-regression analysis of illness severity, we could not use all seven studies, as two studies did not report illness duration and these variables were not available from the authors. Instead, we used five studies for meta-regression analysis of illness duration.

## Results

### Included studies and sample characteristics

**Figure 1:**
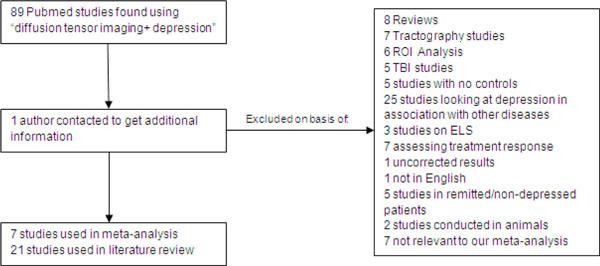
**Summary of exclusion criteria used in finding suitable studies to be included in our meta-analysis**. Seven studies were selected to be included in the meta-analysis based on extensive exclusion criteria as outlined in Methods.

As shown in Figure 1, an exhaustive database search conducted using the key words 'diffusion tensor imaging + depression' resulted in 89 relevant publications. Of these, 82 studies had to be excluded on the basis of being literature reviews (*n *= *8*), using tractography (*n *= 7) or ROI analysis (*n *= 6), studying depression in relation to TBI (*n *= 5), ELS (*n *= 3) or in association with other diseases (*n *= 25), assessing functional or structural response to treatment (*n *= 7), using uncorrected results (*n *= 1) or no controls (*n *= 5), not being available in English (*n *= 1), being conducted in remitted or nondepressed patients (*n *= 5), being conducted in animals (*n *= 2) or not being relevant for other reasons (*n *= 7). Contact with three researchers was made to receive additional required information, but one of the studies was later excluded from our meta-analysis as it used ROI analytical techniques [23].

This resulted in seven high-quality data sets' being selected for inclusion in the meta-analysis, all seven of which were conducted in patients with MDD and none of which were conducted in late-life individuals with MDD, as these latter studies were deemed to be unsuitable for other reasons. Combined the studies included 188 patients with MDD and 221 healthy controls. The original studies were already well matched in terms of age and sex, and no relevant differences were found between patients and controls. Illness severity was calculated by using different scoring systems in the various studies, so an electronic inventory comparing these different scoring systems was used to allow for comparisons between studies (Inventory of Depressive Symptomatology (IDS) and Quick Inventory of Depressive Symptomatology (QIDS); http://www.ids-qids.org/index2.html#table2). Using this inventory, we found that four of the studies were conducted in patients with severe depression, two in patients with moderate depression and one in patients with mild depression. Further details of each of the included studies, such as the presence of anxiety, medication status and duration of illness, are included in Table 1.

### Regional differences in WM FA values

**Figure 2:**
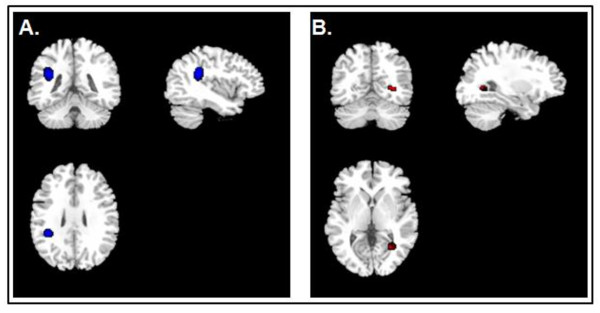
**Signed Differential Mapping-generated MRIcron map of the areas of the brain showing fractional anisotropy alterations in association with depression**. **(A) **Fractional anisotropy (FA) regional differences in patients compared to controls in right fronto-occipital fasciculus. **(B) **FA regional differences in patients compared to controls in left superior longitudinal fasciculus.

The results from the SDM analysis were converted into brain maps and visualised using MRIcron software which were then cross-compared to a Talairach map to optimally localise the brain regions most likely involved (White Matter Atlas; http://www.dtiatlas.org/). Coordinates for the SDM meta-analysis were obtained from all of the seven studies as shown in Table 2 and Figure 2. Patients with depression had significantly smaller FA values in the left superior longitudinal fasciculus (SLF) of the inferior parietal lobe as well as larger FA values in the right inferior fronto-occipital fasciculus (FOF) of the temporo-occipital region.

**Table 2:** Significant regional differences in fractional anisotropy values of the left superior longitudinal fasciculus and right inferior fronto-occipital fasciculus were found in patients with major depressive disorder compared to controls^a^

Region	Talairach coordinates	SDM value	Uncorrected *P *value	Voxel number	Breakdown (number of voxels)
Left SLF, inferior parietal lobe	-38, -38, 26	-0.271	0.00023	179	Inferior parietal lobe (63) Subgyral parietal lobe (70) Insula (25) Supramarginal gyrus (12) Extranuclear (6) Subgyral frontal lobe (1) Superior temporal gyrus (2)
Right inferior fronto-occipital fasciculus	30, -56, 2	0.125	0.000097	33	Subgyral occipital lobe (8) Subgyral temporal lobe (11) Lingual gyrus occipital lobe (4) Middle occipital gyrus (4) Extranuclear (5) Middle occipital gyrus (1)

^a^SDM, Signed Differential Mapping; SLF, superior longitudinal fasciculus.

### Descriptive analysis of quartiles

Large decreases in FA were detected in the SLF in the first quartile analysis (maximum at Talairach -38, -38 and 30; SDM = -0.746, including several nearby clusters). In the median analysis, these decreases were also detected (maximum at Talairach -30, -44 and 22; SDM = -0.159), indicating that in most of the studies we had found some decrease in FA values in this region. Increases in FA were not detected in the quartile analysis, which was related to the fact that only one study showed an increase in FA [24].

### Sensitivity analysis

Whole-brain jackknife sensitivity analysis detected that decreased FA values in the left SLF inferoparietal lobe were present in six of the seven possible combinations and that five jackknife tests showed the posterior part of the SLF, whereas when the study from Wu *et al. *[25] was excluded, the left SLF (temporal) was found. When the study of Jia *et al. *[26] was excluded, the left posterior limb of the internal capsule was detected. Increased FA in the right FOF was reserved throughout six of the seven studies; however, when the study of Blood *et al. *[24] was excluded, this effect disappeared, which we anticipated as it was the only study that reported an increase in FA values. Overall, the results showed a reduced FA in the left SLF to be very stable, with increases in the right FOF being driven by just one study.

#### Linear regression analysis models

**Figure 3:**
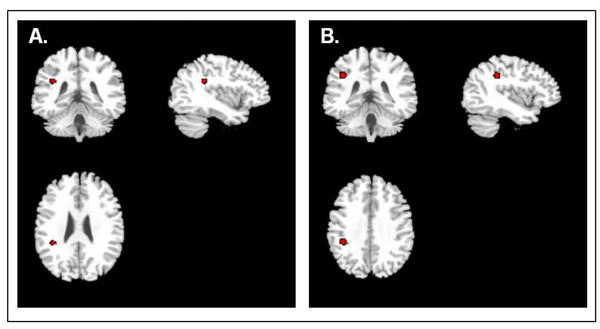
**Reduced FA correlates with increased severity and duration of illness**. **(A) **Reduced FA values were seen in the areas highlighted in red in association with increased severity of depression. **(B) **Reduced FA values were seen in the areas highlighted in red in association with increased duration of illness.

**Figure 4:**
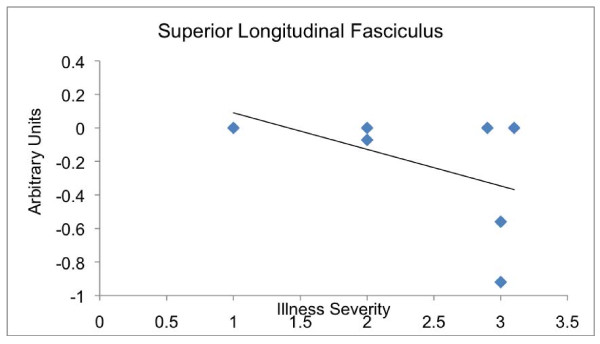
**Correlation between superior longitudinal fasciculus FA values as determined by Signed Differential Mapping (SDM) and illness severity (SDM = -0.396, *P *= 0.000168)**.

Regression analysis showed that depression severity was associated with decreased FA values in the left SLF: Talairach (-38, -38 and 26; five voxels), SDM = -0.396 per one point increase in severity scale (scale of 1 to 3), *P *= 0.000168 (Figures 3A and Figure 4). It also showed an increase of FA in the right FOF: Talairach (26, -54 and 0; 34 voxels), SDM = -0.344 per one point increase in severity scale (scale of 1 to 3), *P *= 0.000017. Moreover, decreased FA values were associated with longer duration of illness in the SLF (-40, -36 and 34; 33 voxels), SDM = 0.087, *P *= 0.0001 (Figure 3B). An interaction analysis showed that researchers in studies including just untreated patients reported significant FA decreases compared to studies with treated patients in the left SLF: Talairach (-38, -38 and 28; 9 voxels), SDM = 0.416, *P *= 0.00077. Moreover, studies with treated subjects had significantly larger FA in the right FOF compared to studies without treated subjects: Talairach (22, -56 and -6; 84 voxels), SDM = 0.478, *P *= 0.00018.

## Discussion

The *Diagnostic and Statistical Manual of Mental Disorders, Fourth Edition *(DSM-IV) [27] defines MDD as the persistence of a depressed mood or loss of interest and pleasure (anhedonia) in association with at least four of the following symptoms: inattention, fatigue, self-depreciation or suicidal thoughts, and disturbances in sleep, appetite, psychomotor activity and weight. The annual incidence of MDD ranges from 12% in women to 7% in men [28], with the WHO ranking MDD as the second leading cause of disability-adjusted life-years in the age category 15 to 44 years for both sexes combined [29]. There is great interest in finding associations between the risk, severity and prognosis of this disease in response to specific changes in certain regions of the brain.

### The relationship between FA alterations and depression varies according to different studies

Seven studies were included in the meta-analysis, resulting in a reasonable sample size (Table 3). Our meta-analysis showed significantly decreased FA values in the SLF in patients with MDD compared to healthy subjects. This effect was significantly more pronounced in studies that included only untreated patients compared to those that included treated patients. FA values in the SLF were also smaller in studies that included patients with a longer illness duration and more severe depression. Studies that consisted solely of untreated patients were also the studies that reported the most severe depressive symptoms. Therefore, it is difficult to distinguish whether this effect is due to treatment or to a more severe depressive illness. With respect to the illness duration, however, there was no difference between treated and untreated patients. Longer, more severe, untreated depressive episodes seemed to be associated with larger FA decreases in the SLF. In the studies that were included in the meta-analysis using DTI brain atlases, the coordinates of significant FA changes were within the SLF region in four of the seven studies, which is in line with our findings [18,24-26]. Interestingly, a recent study identified a reduction of the SLF in healthy adolescents at familial risk for unipolar depression compared to healthy subjects, which provides further support for the previously underestimated importance of SLF in depression [30].

**Table 3:** Summary of diffusion tensor imaging studies included in our meta-analysis conducted in patients with major depressive disorder using corrected whole-brain voxel-based analysis^a^

Study	Method	Participants	Results	Comments
Kieseppä *et al. *[18]	Voxel-based analysis 1.5 Tesla 12 non-colinear directions	16 middle-aged MDD patients and 20 controls	Reduced FA in the left sagittal stratum, cingulate cortex and posterior corpus callosum	Reduced FA in left SLF (temporoparietal) and in the right cingulum in patients with MDD
Zhu *et al. *[58]	TBSS 1.5 Tesla 13 non-colinear directions	25 young first-episode MDD patients and 25 matched controls	Reduced FA in the left anterior limb of the internal capsule, right parahippocampal gyrus and left posterior cingulate cortex	FA values in the left anterior limb of the internal capsule and right cingulum (hippocampus) and left cingulum (dorsal part) were negatively correlated with symptom severity
				
Abe *et al. *[59]	Voxel-based analysis 1.5 Tesla 6 non-colinear directions	21 young MDD patients and 42 controls	No significant difference between groups for FA and WM volume	Negative correlation between FA and total days depressed in both the right anterior cingulate and the left frontal WM
Blood *et al. *[24]	Voxel-based analysis 3.0 Tesla 6 non-colinear directions	22 MDD patients and 22 controls	Increased FA in the right ventral tegmental area and reduced FA in DLPFC WM in MDD subjects	Increased FA in right corticospinal tract, decreased FA in left corticospinal tract, right inferior FOF (frontal lobe), bilateral DLPFC, left SLF (temporal)
Wu *et al. *[25]	Voxel-based analysis 3.0 Tesla 13 non-colinear directions	23 single-episode, medication-naive MDD subjects and 21 controls	FA reduction in right SLF, right middle frontal and left parietal WM with MDD	Reduced FA in left SLF (parietal lobe) and right frontal WM with MDD
Jia *et al. *[26]	Voxel-based analysis 3.0 Tesla 15 non-colinear directions	52 MDD patients, 16 with and 36 without a history of suicide attempts, and 52 matched controls	Reduced FA in the left anterior limb of the internal capsule in suicide attempters relative to both nonattempters and controls	Reduced FA in bilateral SLF (parietal lobe) and cerebellum
				
Korgaonkar *et al. *[60]	Voxel-based analysis 3.0 Tesla 42 different diffusion orientations	29 MDD subjects and 39 controls	7.8% reduction in FA in the limbic system, DLPFC, thalamic projection fibres, and corpus callosum	Limbic-DLPFC-thalamic axis Dysfunction may be involved in MDD

^a^MDD, major depressive disorder; FA, fractional anisotropy; SLF, superior longitudinal fasciculus; TBSS, tract-based spatial statistics; FOF, fronto-occipital fasciculus; DLPFC, dorsolateral prefrontal cortex; WM, white matter.

The association between smaller FA values and illness duration is consistent with findings that show that brain structures decline in volume during ongoing depression [31] and with studies showing a negative correlation between cumulative illness duration and hippocampal volume [32,33]. Aside from the studies included in our meta-analysis, nine additional DTI studies were carried out: seven in late-life depression, six using ROIs and two using tractography analysis (Table 4). Prefrontal regions, the cingulate cortex and the temporal lobe areas were the most commonly used ROIs in these studies. The studies in late-life depression consistently reported reduced FA values in prefrontal brain regions [10,17,34-37]. Reduced FA in the cingulate cortex was also detected [23,36,38]. In addition, Yang *et al. *[17] and Murphy *et al. *[36] reported a reduction in FA values in the parahippocampal gyrus. Aside from the study by Nobuhara *et al. *[10], posterior brain regions were not analysed in these studies. Interestingly, in a combined tractography and voxel-based morphometry study, Cullen *et al. *[38] found reduced FA in connections between the subgenual anterior cingulate cortex to amygdala in the right hemisphere and between the right and left uncinate to supragenual cingulum. However, the sample size was small, with 14 patients and 14 controls. On the other hand, using tractography analysis, Lu *et al. *[39] detected an increase in FA values in patients with MDD in areas of the brain associated with mood regulation, such as the right superior frontal gyrus to right pallidum and the left superior parietal gyrus to right superior occipital gyrus.

**Table 4:** Diffusion tensor imaging studies excluded from meta-analysis conducted in patients with both major depressive disorder and late-life depression (age >65 years) using other forms of analysis such as regions of interest or tractography^a^

Study	Method	Participants	Results	Comments
Lu *et al. *[39]	Tractography 1.5 Tesla 13 non-colinear directions	23 MDD patients, 24 controls	Increased neural connections between areas involved in mood regulation in MDD subjects, such as right superior frontal gyrus to right pallidum and left superior parietal gyrus to right superior occipital gyrus	Increased neural connections in MDD patients in brain areas associated with mood regulation
Cullen *et al. *[38]	Tractography and voxel-based analysis 3.0 Tesla 30 non-colinear directions	14 healthy MDD adolescents, 14 controls	Reduced FA in WM tracts connecting subgenual ACC to amygdala in right hemisphere and right and left uncinate to supragenual cingulum	Altered frontolimbic neural pathways in adolescent depression
Shimony *et al. *[37]	ROI analysis superficial: superior, middle and inferior frontal gyri, medial and lateral orbital frontal, dorsal, ventral and ACC, mesial frontopolar cortex, motor cortex, medial temporal gyrus, fusiform gyrus, auditory cortex, somatosensory cortex and posterolateral intraparietal sulcus, occipital pole and visual cortex Deep: ventral, dorsal, and posterior frontal lobes, temporal and parietal lobes 1.5 Tesla 6 directions	73 LLD subjects, 23 controls	Reduced FA in prefrontal regions compared to controls	DTI abnormalities may be correlated with reduced cognitive processing speed
Yang *et al. *[17]	ROI analysis: DLPFC, parahippocampal gyrus and genu and body of corpus callosum 1.5 Tesla 25 diffusion-weighted directions	31 LLD subjects, 15 controls	Significantly reduced FA values in superior and middle frontal gyrus and right parahippocampal gyrus	Microstructural abnormalities in temporal and frontal areas of brain are associated with LLD
Nobuhara *et al. *[10]	ROI analysis: frontal WM 8 mm above AC-PC, on AC-PC, 8 mm below AC, genu and splenium Bilaterally: temporal WM, parietal WM and occipital WM 1.5 Tesla 6 non-colinear directions	13 LDD patients, matched controls	Significantly reduced FA in the WM of frontal, temporal and occipital brain regions and corpus callosum of LLD individuals	Frontal, temporal and orbitofrontal WM deficits may play a role in symptom severity in LLD
Li *et al. *[35]	ROI analysis: prefrontal WM at 4 mm inferior to and at 0, 4, 8, 12, 16 and 20 mm superior to AC-PC plane 1.5 Tesla 13 non-colinear directions	51 LDD individuals	Significantly reduced FA values in prefrontal WM at bilateral 20 mm, right 16 mm and right 12 mm above AC-PC. No significant correlation between FA and illness course or severity	Prefrontal WM abnormalities may occur early in the course of MDD and may play a role in the pathophysiology
Murphy *et al. *[36]	Voxel-based analysis 1.5 Tesla 8 diffusion sensitization directions	51 LDD individuals, no controls	Reduced FA in WM lateral to ACC and posterior cingulate cortex and in prefrontal, insular and parahippocampal regions	Alterations in the frontostriatal-limbic networks may be associated with executive dysfunction of LLD as measured on the Stroop task
				
Bae *et al. *[34]	ROI analysis: WM of superior and middle frontal gyri of DLPFC, anterior corpus callosum and anterior limb of internal capsule 1.5 Tesla 6 diffusion-weighted directions	106 LLD subjects, 84 elderly euthymic controls	Significantly reduced FA in WM of the right ACC, bilateral superior frontal gyri, and left middle frontal gyrus	Reduced FA in dorsolateral prefrontal cortex and ACC suggests altered brain connections are associated with LLD
Taylor *et al. *[16]	ROI analysis bilaterally: periventricular WM, frontal WM, anterior parietal WM, posterior parietal WM and caudate thalamus 1.5 Tesla 6 non-colinear directions	29 LLD patients and 20 controls	Depressed nonremitting subjects showed fewer changes in ACC	Nonremitting subjects showed fewer FA changes, which may reflect antidepressant failure

^a^MDD, major depressive disorder; FA, fractional anisotropy; WM, white matter; ROI, region of interest; LLD, late-life depression; DTI, diffusion tensor imaging; DLPFC, dorsolateral prefrontal cortex; ACC, anterior cingulate cortex; AC, anterior commissure; PC, posterior commissure.

### FA changes occurring in individuals exposed to ELS may underlie subsequent psychiatric sequelae

DTI studies conducted in individuals exposed to high levels of ELS commonly show reduced FA values compared to controls matched for age and gender (Table 5). Paul *et al. *[19] showed a significant reduction in the genu of the corpus callosum in women subjected to high levels of ELS compared to controls matched for age, gender and education and these changes were also seen in the absence of psychiatric symptoms. A study by Choi *et al. *[40] reported a significant decrease in FA in the left superior temporal gyrus in association with parental verbal abuse (PVA), whereas Tomoda *et al. *[41] reported a 14.1% increase in the grey matter of this area. Despite their conflicting evidence, both studies concluded that PVA causes alterations in the neural pathways responsible for language processing and development. A ROI and tractography study conducted in seven socioeconomically deprived children and controls found significant reductions in FA values of the uncinate fasciculus and suggested these changes might underlie the cognitive and behavioural changes that occur in children exposed to high levels of ELS [42]. A study of the effects of posttraumatic stress disorder (PTSD) in children reported reduced FA in the medial and posterior corpus callosum compared to controls matched for age, sex, race, IQ and handedness, areas of the brain involved in emotional processing [43].

**Table 5:** Diffusion tensor imaging studies conducted in individuals exposed to high levels of early-life stress reporting fractional anisotropy alterations^a^

Study	Method	Participants	Results	Comments
Paul *et al. *[19]	ROI analysis 1.5 Tesla 12 non-colinear directions	116 healthy subjects older than 18 years of age	Reduced FA within genu of corpus callosum in female subjects only who were exposed to significant ELS	ELS causes microstructural changes in brain that also occur in absence of clinically significant psychiatric symptoms
				
Eluvathingal *et al. *[42]	ROI and tractography 1.5 Tesla 6 non-colinear directions	Seven right-handed children with history of early, severe socioemotional deprivation, seven matched controls	Significantly reduced FA values in left uncinate fasciculus in individuals exposed to ELS compared to controls	Structural changes in left uncinate fasciculus may contribute to cognitive and behavioural problems seen in ELS children
Choi *et al. *[40]	TBSS 3 Tesla 12 encoding directions	16 unmedicated individuals with history of high-level exposure to PVA but no other form of maltreatment, 16 matched controls	Reduced FA in arcuate fasciculus in left superior temporal gyrus, cingulum bundle by posterior tail of left hippocampus and left body of fornix	PVA exposure may cause alterations in integrity of neural pathways with consequences for language development and psychopathology
Tomoda *et al. *[41]	Voxel-based whole-brain analysis 3 Tesla	21 unmedicated, right-handed subjects ages 18 to 25 years with histories of PVA, 19 controls	An increase in grey matter volume by 14.1% in the left superior temporal gyrus	PVA exposure may affect development of auditory association cortex involved in language processing
Jackowski *et al. *[43]	ROI analysis 1.5 Tesla 6 non-colinear directions	17 maltreated children with PTSD, 15 controls	Reduced FA in medial and posterior corpus callosum of maltreated children	Alterations in areas of brain important in processing of emotional stimuli and memory function is associated with ELS

^a^ELS, early-life stress; ROI, region of interest; FA, fractional anisotropy; TBSS, tract-based spatial statistics; PVA, parental verbal abuse; PTSD, posttraumatic stress disorder.

Environmental factors experienced during the early neonatal period appear to have powerful and enduring influences on an organism's physiology and behaviour [44]. Childhood abuse has been associated with increased rates of depression, anxiety, suicide, panic disorder, PTSD, attention-deficit/hyperactivity disorder and other behavioural disorders [45,46]. A community-based study of 1,931 women found larger increases in the rates of depression, anxiety and suicide attempts in women with a history of childhood physical and sexual abuse than in women who were victims of rape and assault in adulthood [47]. The psychiatric sequelae seen in adulthood have been linked to structural changes that occur in the brain secondary to increased levels of stress hormones that result from abuse [48]. Childhood abuse and neglect lead to a decrease in cell proliferation and neurogenesis in the dentate gyrus of the hippocampus in adult brains [49]. This inhibition of neuroplasticity caused by glucocorticoid hypersensitivity leads to diminished stress coping mechanisms in adulthood, conferring a higher risk for the development of MDD [49]. Numerous preclinical studies have shown that ELS induces long-lasting hyperactivity of corticotropin-releasing hormone (CRH) with subsequent increased levels of cortisol and corticosterone as well as alterations in other neurotransmitter systems [46]. A number of studies conducted in children who experienced ELS at different developmental stages found increases in salivary cortisol levels as well as a loss in the normal circadian rhythm of cortisol secretion, suggesting a persistent sensitization of stress-responsive neural circuits [46].

### Biological basis for the microstructural alterations seen in MDD

#### The role of the immune system

Cytokines such as IL-1, IL-6 and TNF-α play important roles in peripheral inflammation, provide neurotrophic support and enhance neurogenesis in the central nervous system and contribute to normal cognitive processes such as memory formation in laboratory animals [50]. In contrast, reduced neurogenesis, increased glutamatergic transmission, increased oxidative stress, glial apoptosis and dysregulation of neuronal and/or glial interactions have all been reported when these cytokine levels become chronically elevated [50].

#### The role of hypothalamic-pituitary-adrenal axis dysfunction

Hypothalamic-pituitary-adrenal (HPA) axis dysfunction is a common finding in both human and animal models of MDD [49,51,52], and restoration of normal function is a prerequisite for effective MDD treatment [5]. This dysfunction is believed to be due in part to hyperactive CRH neurons, which leads to overactivity of the HPA axis and produces symptoms of anxiety and depression [53]. The resultant increase in glucocorticoid secretion leads to an increase in excitatory amino acid neurotransmitters such as glutamate, with initial reversible remodelling and eventual cell death in the hippocampus, a highly stress-sensitive area of the brain [4]. Glucocorticoids also eliminate activity-dependent increases in brain-derived neurotrophic factor, a growth factor that is important in the formation of neural connections and inhibits dendritic branching in response to stimuli [4].

#### The role of epigenetics

'Epigenetics' refers to the diversity in gene regulation that occurs during development and alters an organism's behavioural and physiological responses to its environment [54]. Epigenetic regulation of the glucocorticoid receptor (GR) gene may have an important role in predisposing an individual to developing MDD and has been proposed as a mechanism involved in stress-induced neural toxicity [51]. Weaver *et al. *[55] reported that increased maternal rat pup-licking, pup-grooming and arched back pup-nursing influenced hippocampal GR expression and thus HPA axis function through epigenetic regulation of NGFI-A binding to the exon 17 promoter. Importantly, this was reversed by cross-fostering pups from low-maternal-care mothers to high-maternal-care mothers and *vice versa*, suggesting that this alteration in HPA axis regulation was not hereditary and was due purely to environmental epigenetic regulation [55]. Changes in the epigenetic status of the GR gene were also observed by MacGowan *et al. *[54] in response to alterations in parental care during the early postnatal period in rat pups. In a study of suicide victims with or without a history of child abuse as well as healthy controls, hypermethylation of the GR gene was found only in individuals with a history of child abuse [51]. Recently, we demonstrated that subjects carrying the risk short (S) allele of the polymorphism in the promoter region of the serotonin transporter 5-HTTLPR had smaller hippocampal volumes when they had a history of ELS compared to subjects who only carried the genetic risk or compared to those who had a history of ELS but did not carry the risk S allele [56]. This suggests that epigenetic mechanisms may affect brain structure.

Incredibly, new evidence exists that suggests that increased resilience to depression may be due to stronger neural connections in the prefrontal or orbitofrontal, temporal and parietal areas of the brain [9]. A study comparing 21 healthy relatives of MDD patients to 24 healthy volunteers without such a family history found higher FA values in these brain regions in individuals with a positive family history [9]. As a family history of MDD normally confers a higher risk of developing this disease [5,53], these findings suggest that individuals who manage to stay healthy have increased resilience in the form of stronger neural connections (as indicated by higher FA), which may be secondary to epigenetic regulation [9].

None of the nine excluded studies reported significant FA alterations in the SLF or FOF, and Li *et al. *[35] reported no correlation between FA values and illness course or severity. Thus these ROI and tractography studies provide some evidence that there may also be changes in diffusivity in tracts connecting prefrontal, cingulate and medial temporal lobe (amygdala and parahippocampal) regions. Also, it is difficult to distinguish the effects of illness duration from the effects of illness severity in our sample, and this is a potential area for further research. Meta-analytical methods need to include strict inclusion criteria to ensure that the studies sampled are of high quality. However, this limits the extrapolation of results, since more naturalistic studies and explorations are not taken into consideration. We tried to overcome this issue by reviewing all other DTI studies that used ROI approaches, tractography or whole-brain voxel analysis without correcting for multiple comparisons in the discussion.

## Conclusion

In conclusion, MDD is a serious and occasionally life-threatening disease that is a result of many complex, interconnected processes occurring in tandem within the brain. Factors such as inherited genotype, epigenetic regulatory processes, developmental experiences and chronic stress all affect the functional and structural integrity of limbic brain regions involved in emotional processing to increase or decrease an individual's vulnerability to depression. Our meta-analysis found decreased WM FA values in the SLF and increased FA values in the FOF. The increase in the FA value of the FOF was driven by only one study, whereas the FA reduction in the SLF was consistently reported throughout all of the various analyses. The SLF is believed to be the major association fibre pathway that connects the parietotemporal association areas with the frontal lobe [57]. Although to our knowledge no previous studies have focused specifically on the relationship between the SLF pathway and depression, our meta-analysis found a significant reduction in the FA value of this tract in association with depression. Indeed, disease variables such as increased severity and duration of illness produced further reductions in FA, suggesting a direct association between disease progression and tract abnormality. Our study further strengthens the concept that microstructural abnormalities as determined by DTI analysis may underpin many of the features of psychiatric illness, in particular depression.

## Competing interests

The authors declare that they have no competing interests.

## Authors' contributions

Both authors made substantial contributions to the study's conception and design, as well as to the acquisition and analysis of data. They were involved in drafting the manuscript and gave their final approval of the version to be published.
